# Comparison of the efficacy of low doses of methylprednisolone, acetaminophen, and dexketoprofen trometamol on the swelling developed after the removal of impacted third molar

**DOI:** 10.4317/medoral.20582

**Published:** 2015-08-04

**Authors:** Cennet-Neslihan Eroglu, Hanife Ataoglu, Gulsun Yildirim, Demet Kiresi

**Affiliations:** 1 Department of Oral and Maxillofacial Surgery, Yuzuncu Yil University Faculty of Dentistry, Van, Turkey; 2 Department of Oral and Maxillofacial Surgery, Selcuk University Faculty of Dentistry, Konya, Turkey; 3 Department of Radiology, Konya Necmettin Erbakan University Meram Faculty of Medicine, Konya, Turkey

## Abstract

**Background:**

The aim of the present study was to compare the efficacy of low doses of methylprednisolone, acetaminophen and dexketoprofen trometamol, which are among the drug groups used in our clinic, on postoperative swelling developing after removal of impacted third molar.

**Material and Methods:**

The three group of patients received either 40 mg methylprednisolone or 300 mg acetaminophen or 12.5 mg dexketoprofen trometamol one hour before the procedure, according to the patient groups. The patients in the methylprednisolone group were injected with methylprednisolone at a dose of 20 mg 24 hour after the procedure and prescribed 300 mg acetaminophen as rescue analgesic. During the postoperative period, the doses that were given before the procedure were continued 3 times a day for 2 days in the acetaminophen and dexketoprofen trometamol groups. Maximal swelling was assessed preoperatively and at the postoperative 48 hours by ultrasound images.

**Results:**

Swelling was 34% lower in the methylprednisolone than in the other groups; however, no statistically significant difference was found between the groups. The acetaminophen and dexketoprofen trometamol groups exhibited clinical results close to each other.

**Conclusions:**

Combination of low doses of methylprednisolone and acetaminophen provide a safe and adequate clinical success on swelling.

**Key words:** Methylprednisolone, acetaminophen, dexketoprofen trometamol, third molar extraction, swelling.

## Introduction

After surgical removal of teeth, daily lives of patients are affected to different extents due to inflammatory response (1). Postoperative edema developing during this inflammatory process, even if short term, may raise esthetic concerns for the patient. Since the development of edema is important in the first 48 hours, this duration is important to prevent edema. After the development of edema, healing occurs in a one-week period. The shortening or elimination of this period gives the patient a great comfort.

The mechanism of the development of postoperative edema is the release of inflammatory mediators, like prostaglandins, leukotrienes, bradykinin and platelet activating factor to the wound region after surgical intervention, and vascular dilatation and permeability (2,3).Therefore, based on this, for decreasing vascular permeability that leads to acute inflammation and for reducing fluid transfer between tissues, various methods such as the use of non-steroid anti-inflammatory drugs (NSAIDs), various steroids, antihistaminic drugs, and enzymes, placement of drains, low level laser therapy, and ice therapy have been used (2,4-6).

Non-steroid anti-inflammatory drugs have been the most commonly used drug group in the postoperative period due to their practical use and wide availability. In the literature, there are numerous studies showing that these drugs, besides their analgesic properties, prevent or reduce postoperative edema. Dexketoprofen trometamol (DT) is a ketoprofen derived NSAID (7). The analgesic efficacy of DT, which is thought to affect inflammation by the inhibition of cyclooxygenase activity, has been widely studied in clinical trials, although sufficient emphasis has not been laid on its anti-inflammatory properties.

Acetaminophen, also called paracetamol, is a para-aminophenol derivative non-opioid drug, with central analgesic and antipyretic effects (8). It is a weak inhibitor of cyclooxygenases in the peripheral tissues, and as it does not quite affect prostaglandin synthesis that has a great effect on the development of inflammation, some authors claim it has no anti-inflammatory effects, while some suggest that it has some anti-inflammatory properties (9-11). Corticosteroids are the most commonly preferred drugs among pharmaceutical agents by the clinicians for preventing and reducing edema. Corticosteroids show anti-inflammatory effects by inhibiting phospholipase A2 activation that provides the release of arachidonic acid into the inflammation region (phospholipase A2 enzyme stimulates prostaglandins, leukotrienes, and neutrophil migration and causes inflammation) (12). Methylprednisolone is also a corticosteroid reducing macrophage infiltration in the inflammation region and the number and proliferation of fibroblasts in the connective tissue; additionally it suppresses the immune system (13). Among the mentioned edema prevention methods, there is no standard for doses of drugs. Based on the clinical studies ranging from using single drug dose to very high doses, and taking into account the side effects of the drugs, no optimal dose range could be determined.

Therefore, this single blind study was planned to determine the maximum clinical effect of methylprednisolone, acetaminophen and DT on edema, keeping the repeat doses at minimum.

## Material and Methods

Forty-five outpatients between 18 and 35 years of age, who attended Selcuk University Faculty of Dentistry the Department of Oral and Maxillofacial surgery, requiring surgical removal of unilateral impacted mandibular third molar teeth under local anesthesia, were included in the study. The teeth were Class II, Class B according to Pell-Gregory and mesioangular according to Winter classifications. Bone removal and tooth sectioning were necessary in all operations. The study protocol and the informed consent forms were approved by the Research and Ethics Committee of the Yuzuncu Yil University, Faculty of Medicine (YYU-19062014/11). The study protocol was explained to the patients in detail after which consent was obtained. Patients with good oral hygiene, no contraindication to use corticosteroids, analgesics, or local anesthetics, without any systemic diseases and no drug intake within 24 hours of the operation were included. All patients underwent clinical examination and panoramic radiographs were taken. All operations were performed by the same surgeon in an effort to minimize differences due to operator variability, using a standardized technique under local anesthesia. In all surgical interventions, mucoperiosteal flap and osteotomy were performed. For maintenance of anesthesia, 4% articaine HCl with 1:100.000 epinephrine was used.

Patients were randomly allocated into three groups. In the methylprednisolone group, each patient was given an intramuscular injection (gluteal region) of 40 mg methylprednisolone (Prednol-L, Mustafa Nevzat Drug Industry, Istanbul, Turkey) one hour before the operation and 20 mg methylprednisolone 24 hours after the removal of the impacted third molar. Patients in the methylprednisolone group were additionally given 300 mg acetaminophen (Panalgine, Atabay Drug Industry, Istanbul Turkey) every 8 hours post operatively. In the acetaminophen group, 300 mg acetaminophen (Panalgine, Atabay Drug Industry, Istanbul, Turkey) was given one hour before the surgery and 300 mg acetaminophen was continued post operatively at every 8 hours for a minimum of two days. The DT group was administered 12.5 mg DT (Arveles, Ufsa Drug Industry, Istanbul, Turkey) one hour before the operation and continued on 12.5 mg DT every 8 hours post operatively for two days. All patients were given chlorhexidine mouthwash two times a day for seven days starting from the operation.

Each patient was evaluated preoperatively and 48 hours post operatively by using ultrasound for facial swelling. The thickness of the soft tissue at the masseteric region was measured. For this step, patients were referred to the Radiology Department of Necmettin Erbakan Medical Faculty. All examinations were performed by the same blinded radiologist and same ultrasound device (Logiq 9, GE, USA) with 7.5 MHz linear array transducer). Ultrasound examinations were performed in the supine position. For evaluation of facial swelling, ultrasound probe was placed parallel to the occlusal plane and perpendicular to mandibular ramus. Swelling was evaluated with preoperative and postoperative ultrasound images; the difference between the preoperative and postoperative measurements indicated the size of swelling.

Statistical analysis of the study was performed using software program SPSS (version 21) for Windows. Kruskal-Wallis test was used. A *p* value <0.05 was considered statistically significant.

## Results

The study was started with 45 patients; however, nine patients were eliminated because of failure to return for ultrasonographic evaluation. Of 36 patients, who successfully completed the trial, 13 were men and 23 were women, with a mean age of 21.83 years. The mean operative time from the initial incision to the final suture was recorded as 16.32 min.

Statistical analysis of the data indicated no differences between the groups with regard to swelling ( Table 1). Although there was no significant difference between the groups, the effect of methylprednisolone plus acetaminophen was also evident on peak swelling, which was defined as the maximal swelling observed at the second day after surgery. According to the clinical results, peak swelling was markedly less in the methylprednisolone group than that in the acetaminophen and DT groups. Clinically, the highest swelling was observed in the DT group. No significant difference was found between the female and male patients in terms of postoperative swelling ( Table 2). Moreover, no significant difference was determined among the groups in terms of age ( Table 2).

**Table 1:**
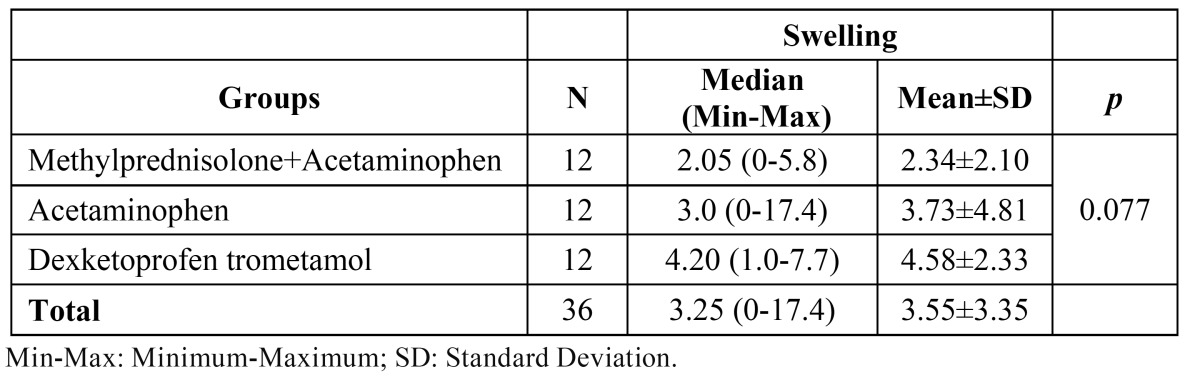
Descriptive statistics for swelling in the groups and comparison results.

**Table 2:**
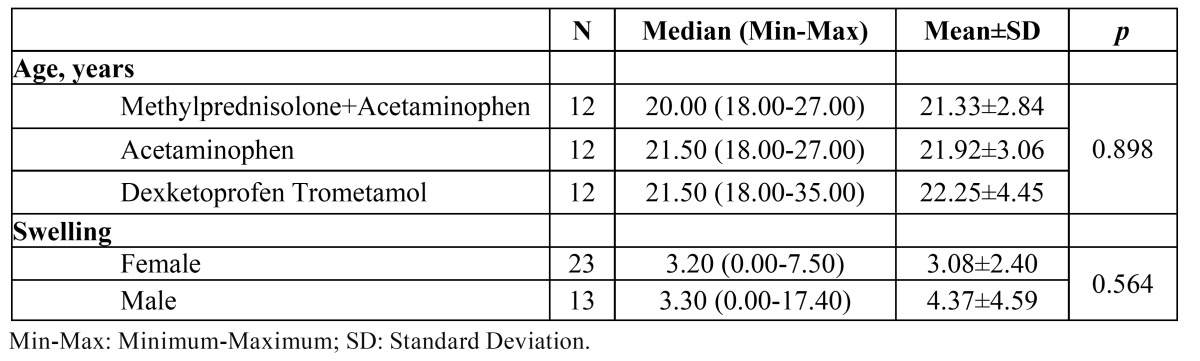
Age of the study groups and swelling according to gender and comparison results.

None of the patients experienced any adverse effects that could be attributed to drugs. However, two patients in the methylprednisolone group developed alveolar osteitis.

## Discussion

Although there are studies evaluating the effect of different doses of various drugs on postoperative complications in the literature, we, in this study, examined the efficacy of different drug groups used in routine practice on postoperative edema with low repeated doses in order to keep the side effects at minimum.

This present study is important from the clinical standpoint in terms of safe doses of commonly used drug groups in the postoperative period.

In the preference of the suitable corticosteroid according to patient and procedure, it is quite important to prefer a drug with minimal mineralocorticoid activity and minimal effect on biological activity (14). As the duration of action and half-life (18 to 36 hours) of methylprednisolone is long and as it has almost no mineralocorticoid activity (14,15), we preferred to use methylprednisolone in this study. As known, although corticosteroid use may lead to various unwanted effects, short-term use in healthy individuals does not carry a risk for treatment delay and local infection (16). Corticosteroid use is among the etiological factors of postoperative alveolar osteitis (17). Nevertheless, a study has shown that oral steroid use has no effect on alveolar osteitis (18). Considering that short-term low dose corticosteroid was used in the present study, it may be incorrect to attribute the development of alveolar osteitis in two patients in the methylprednisolone group to corticosteroid use. It was thought that these cases of alveolar osteitis might develop due to patient-related factor.

Studies on the removal of impacted tooth claim that preoperative single dose intramuscular use of 40 mg methylprednisolone is effective on inflammatory response (19,20). However, when half-life of methylprednisolone and the time that postoperative maximal swelling is observed are taken into account, a preoperative single dose may not be adequate.

In the literature, in a study that used a lower dose of methylprednisolone, 20 mg, by oral, IV, and IM route and evaluated the relation with anti-inflammatory effect, no statistically significant relationship was found; however, it was observed that intramuscular application is more successful clinically (21).

Although a standard dose could not be suggested based on the previous studies on prevention of postoperative complications using methylprednisolone, the most frequently used dose is 40 mg (19,20,22,23). There are also studies in which methylprednisolone doses ranging from 15 mg up to 125 mg have been used (13,21,24-26). In the study by Vegas-Bustamente *et al*. (19), 40 mg of single dose methylprednisolone injected into the masseter muscle after the extraction of impacted lower third molars was found to be effective on pain, trismus, and swelling. In their study, Micó-Llorens *et al*. (20) determined that single dose methylprednisolone injected via IM route just after surgical extraction of impacted lower third molars provided a comfortable postoperative course, particularly for the first postoperative 2 days, in terms of pain, trismus, and swelling. In our study, 40 mg intramuscular methylprednisolone was given as the first dose, a repeat dose of 20 mg methylprednisolone was also applied by the intramuscular route, and the patients in this group continued on 300 mg of acetaminophen as analgesic. Although our aim in the present study was to provide postoperative analgesic support rather than to increase the effects of the drugs, use of corticosteroid and acetaminophen together may have increased their potential effects. Despite there was no statistically significant difference between the groups, the group that demonstrated the minimal postoperative swelling was the methylprednisolone plus acetaminophen group (49% more effective on swelling compared to DT and 37% more effective on swelling compared to acetaminophen). We think that statistical success will reach clinical success, if the number of patients is increased.

Although acetaminophen has not been included in any anti-inflammatory drug group, it has been reported to demonstrate better anti-inflammatory effect at high doses, even better than NSAIDs. Bjørnsson *et al*. (10) compared the effects of 500 mg naproxen sodium, an analgesic NSAID, with 1000 mg acetaminophen in postoperative inflammatory process and found that acetaminophen reduced the swelling by 22.4% in comparison to naproxen. In another study of the same authors, 1000 mg acetaminophen was demonstrated to have preferable anti-inflammatory effects in comparison to 600 mg ibuprofen (11). Nonetheless, there are studies reporting that acetaminophen used at high doses had no preventable effect on swelling (27). According to the results of our study, 300 mg acetaminophen exhibited approximate clinical results with 12.5 mg DT, a NSAID. However, generally speaking, acetaminophen used at these doses does not give well enough results on swelling.

Even though DT has been shown to be a good analgesic by numerous studies, the number of studies on its anti-inflammatory properties is few. Although it has been reported that 25 mg DT has a greater anti-inflammatory effect than 600 mg ibuprofen on swelling after impacted third molar surgery (28), it could not be mentioned about a sufficient anti-inflammatory effect with the half of the DT dose in the present study. Similar to this finding, Eroğlu *et al*. (29) reported that DT at a dose of 12.5 mg was effective as analgesic but had a weak anti-inflammatory effect on swelling and trismus in their study, in which the effects of 12.5 mg DT and 500 mg paracetamol on postoperative complications in bilateral symmetric impacted teeth were compared. Indeed, DT at a dose of 12.5 mg provides almost the same analgesic effect as dipyrone at a dose of 575 mg (30); however, it does not exhibit anti-inflammatory effect at this dose. As 12.5 mg is the safe dose of DT (DT has been reported to have side effects at doses of 25 mg and over, especially in the presence of a systemic disease) (31,32), we preferred to use 12.5 mg DT in this study. Despite its side effects, DT is generally preferred to be used at a dose of 25 mg in the studies (28,33).

The majority of clinical studies are planned to determine the most efficacious and safest analgesic drug effective at the lowest dose possible. Analgesic efficacy of the drug groups we studied have been examined in numerous studies and their analgesic efficacy were confirmed. However, there is limited number of studies investigating the anti-inflammatory effect of these drugs in the literature. Therefore, we evaluated the effects of the drug groups used in routine practice at the lowest doses possible on swelling which makes the patients feel uncomfortable in the postoperative period. Conclusively, the drugs used in the present study at the given doses were well tolerated without any side effects. In patients undergoing oral surgery, the combination of preemptive preoperative methylprednisolone and postoperative acetaminophen was found to be clinically successful on postoperative swelling without any side effects.
